# Prognosis of refractory neurosarcoidosis altered by thalidomide: a case report

**DOI:** 10.1186/1752-1947-2-27

**Published:** 2008-01-28

**Authors:** J Chad Hoyle, Herbert B Newton, Steven Katz

**Affiliations:** 1 Dardinger Neuro-Oncology Center, Division of Neuro-Oncology, and Department of Neurology, The Ohio State University Medical Center and James Cancer Hospital & Solove Research Institute, Columbus, Ohio, USA

## Abstract

**Introduction:**

Sarcoidosis is a multisystem disease characterized by noncaseating granulomas in the lungs, skin, lymph nodes, and, rarely, the nervous system. Granuloma formation in sarcoidosis is mediated by increased secretion of interferon-gamma, interleukin-2, and tumor necrosis factor-alpha. 25% of patients with neurosarcoidosis are steroid resistant and another 20–40% are resistant to any conventional immunosuppression, but the typical agents suppress the immune system in a non-specific fashion. Thalidomide has been shown to have activity specific to the inflammatory mediators of sarcoidosis, has been shown to be beneficial in cutaneous sarcoidosis, and provides an interesting observation in our patient with refractory neurosarcoidosis.

**Case presentation:**

A 40 year old African-american female presented with refractory neurosarcoidosis. Over the course of several years, the patient was treated with high dose steroids, imuran, cytoxan, and cyclosporine without benefit. Then, the patient received thalidomide, slowly escalating to 650 mg. After 2 months radiologic improvement was noted and after 6 months clinical stabilization and improvement became apparent.

**Conclusion:**

Our case report presents a difficult, refractory case of neurosarcoidosis that demonstrates an altered prognosis based on the addition of thalidomide.

## Introduction

Sarcoidosis is a granulomatous, inflammatory disease that most commonly involves the lungs, skin, and eyes, but can also affect the nervous system in about 5% of cases [1]. Though it is common for the disease to spontaneously remit, nervous system involvement is a marker for a more resistant course [2]. Because of this, nervous system involvement is a definite indication for corticosteroid treatment. Unfortunately, 25% will still have a refractory course with steroid treatment and, even more concerning, 20–40% of those refractory patients will not respond to any level of current conventional immunosuppression [1,3].

Despite the identification of several possible candidates, the exact inciting antigen responsible for the formation of sarcoid granulomas remains uncertain. However, the resultant inflammatory cascade involved in mediating granuloma formation has been mapped out more accurately. It involves macrophages or dendritic cells ingesting the antigen with resultant peptide fragments bound to MHC-II complexes that ignite a polarized TH-1 inflammatory cascade. This involves Interleukin-2, Interleukin-12, interferon-gamma (IFN-γ), and tumor necrosis factor-alpha (TNF-α). Specifically, TNF-α has gained clinical attention with the availability of TNF-α blocking agents [4]. The prognostic outcomes have been based on conventional immunosuppression that attacks this process in a non-specific fashion, but we present a case that contradicted a refractory outcome after the patient received thalidomide, which targets the immune cascade in a specific fashion.

## Case Presentation

A 40 year-old african-american female presented with a 3-year history of headaches, episodes of altered awareness, fatigue, back and neck pain, and progressive visual loss. The headaches were the initial complaint and were considered migrainous in origin, but medications failed to alter their building intensity. The progressive visual loss then became more prominent and instigated a thorough work-up.

The vision of the right eye was affected first (20/300 OD, 20/25 OS). A magnetic resonance imaging (MRI) scan of the brain demonstrated bilateral optic nerve involvement, so sarcoidosis was suspected, but a biopsy that included tissue from the sclera, optic nerve sheath, and lacrimal gland unfortunately was non-diagnostic. A chest CT and serum ACE levels were initially normal, and a lumbar puncture (wbc 9, protein 86, glucose 37, positive oligoclonal bands, negative cytology) had a normal ACE level, as well. Though the above testing did not exclude the possibility of sarcoidosis, the lack of definitive findings brought other possibilities into the differential diagnosis. So, in addition to daily prednisone at 60 mg QD initiated at the onset of visual deficits, the patient was also empirically given a one year trial of avonex for the potential diagnosis of "atypical MS." At that time her symptoms consisted of right arm paresthesias and numbness, as well as the aforementioned visual symptoms.

Notwithstanding these efforts, the patients' neurologic status continued to decline, especially the vision in her left eye, which was now becoming more clinically affected (i.e., 20/200). The avonex was discontinued, and the patient was started on imuran (50–100 mg/day), while continuing prednisone (60–80 mg po QOD) over the next 6 months. A repeat chest CT was performed, which now showed grade I hilar adenopathy, so gallium scintigraphy was undertaken, which revealed bilateral increased activity within the hilum, orbits, and mediastinum. Consequently, a lung biopsy was performed, and this confirmed non-caseating granulomatous tissue, and a diagnosis of neurosarcoidosis.

The patient's clinical decline continued, so more aggressive immunosuppression was pursued. The patient received four intravenous steroid pulses and two treatment cycles of intravenous cyclophosphamide. Leukopenia prevented further treatment with cyclophosphamide. However, oral cyclosporine was started at 25 mg po BID to accompany oral prednisone at 60 mg po QOD.

**Figure 1:**
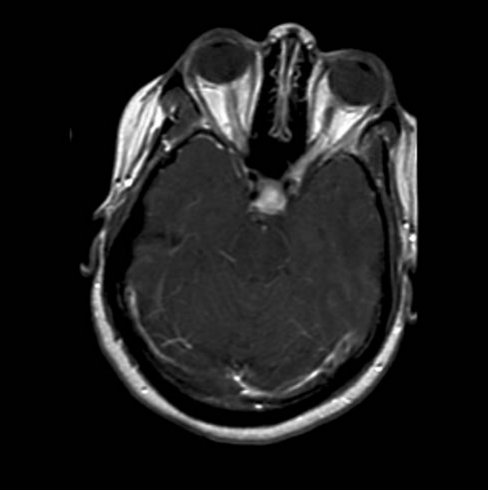
MRI findings one month before initiation of thalidomide, showing marked enhancement of infundibulum (6 mm) and bilateral optic nerves (6 mm).

Throughout the next seven months, while on this more aggressive immunosuppressive regimen, the patient had further deterioration. She lost all functional vision, including light perception. The headaches persisted and the back and neck pain became more severe, with a more prominent radicular component. The patient required a cane for ambulation, and often required the use of a wheelchair, due to the extremity pain and visual loss. In addition, migratory paresthesias became more prominent. The patient's spells of altered awareness were found to be complex partial seizures, and the patient required management with levetiracetam (titrated to 1000 mg tid). Repeat MRI of the brain (see figure 1) had also progressed, now showing more prominent enhancement and thickening of the optic nerves, bilaterally (up to 6 mm in greatest thickness). The thickness of the infundibulum had also increased, up to 6 mm. Overall, meningeal enhancement was more diffuse throughout the brain.

Due to the refractory nature of the neurosarcoidosis, the patient investigated multiple opinions and was referred for a trial of thalidomide. The patient was started on 50 mg po BID of thalidomide with the goal of slowly titrating up to a maximum dose of 800 mg per day. The patient was titrated up to 650 mg per day over a 6 month time period, but due to excessive fatigue and sedation, could not tolerate a higher dose.

**Figure 2:**
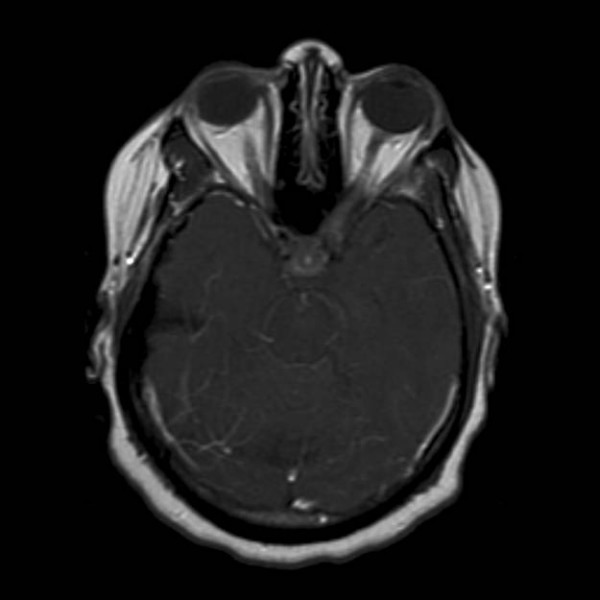
MRI Brain with contrast demonstrating improvement 2 months after initiation of thalidomide.

**Figure 3:**
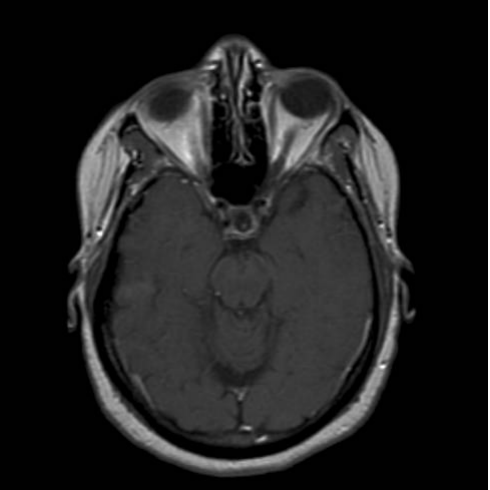
MRI brain with contrast showing near resolution of enhancement, stable on serial MRIs for 2 years.

After two months of treatment, the patient had radiographic improvement on follow-up MRI scan (see figure 2). Following another 4 to 10 months of thalidomide, there was complete resolution of all abnormal enhancement on MRI, which has been maintained over the past several years (see figure 3).

Following six months of therapy, her symptoms began to stabilize and then slowly resolve. The radicular neck and back pain markedly improved, with resultant facilitation of her ambulation. Migratory paresthesias lessened and headaches became much less severe. In addition, the complex partial seizures were stabilized. The only poor outcome was a lack of significant improvement in the vision. Over the next year, cyclosporine was discontinued and prednisone was slowly weaned to 5 mg per day. Throughout this wean, the previously noted clinical improvements remained stable.

The only significant side effect attributed to the thalidomide was sedation, which did limit the peak dose to 650 mg per day, even with a slow titration schedule. Due to the favorable clinical response, following several years of therapy, the dose has slowly been weaned down to 300 mg per day. The patient is stable on this dose, and plans are for further weaning, as tolerated. The patient did have some transient numbness and paresthesias in her feet, but this resolved on the lower dose of thalidomide. She has had no definite evidence of thalidomide neuropathy.

## Discussion

Thalidomide, an immunomodulatory agent has the ability to inhibit all of the inflammatory mediators specific to sarcoidosis, and, particularly, has very potent activity against TNF-α [5]. With its known success in cutaneous sarcoidosis, thalidomide might offer a better outcome for patients with refractory neurosarcoidosis [5]. In this report, we have described a patient that might further corroborate this hypothesis.

Thalidomide was introduced as a hypnotic in the 1940's and was well tolerated until its teratogenic effects were discovered (i.e., phycomelia). It was pulled from the market but gained a resurgence for the treatment of leprosy and other conditions needing significant anti-inflammatory effects [6]. Most importantly, it has shown efficacy against cutaneous sarcoidosis, making it a potential candidate for neurosarcoidosis, as well. Dosages from 100–800 mg per day are used, with a slow titration towards 800 mg, if tolerated [5]. The main side effect that may potentially limit treatment is sedation. Other side effects to be aware of include rash, thromboembolism, dizziness, constipation, and a painless, axonal sensory neuropathy that develops in about 20% of patients. Because of the significant teratogenic concern, thalidomide can only be prescribed if the physician is a member of the STEPS (System for Thalidomide Education and Prescribing Safety) program [7]. Interestingly, there is not an increased risk of infection in patients on thalidomide, which is in contrast to other immunosuppressive therapies used for sarcoidosis [6].

The patient in our case report appeared to respond well to thalidomide without significant side effects, except dose-limiting fatigue and somnolence. This is encouraging and could further bolster experimental use of this medication in refractory neurosarcoidosis, with the eventual possibility of clinical trials.

The patient had progressive clinical symptoms and worsening of MRI findings (see figure 1), with increased enhancement, despite high dose steroids and other aggressive immunosuppressive medications. It is interesting to note that the radiographic progression of enhancing brain lesions despite treatment correlates with a 75% chance of a refractory course, further strengthening the argument that this patient's prognosis was poor [8]. After the initiation of thalidomide, the patient noted a plateau of clinical symptoms within 6 months, and then demonstrated steady continual improvement thereafter, except for the visual loss. The lack of improvement of vision suggested that the optic apparatus (i.e., optic nerves, chiasm, optic tracts) was too severely damaged from sarcoidosis-mediated inflammation (i.e., axonal loss) to allow recovery. The 2 month follow-up scan (see figure 2) after thalidomide was started, as well as subsequent scans (see figures 3) after that, convincingly showed improvement until resolution of the enhancement 6–12 months later.

Arguments against thalidomide providing this benefit include that the disease naturally burned itself out or that the conventional immunosuppressive agents did not have enough time or high enough dosages to provide benefit. Though it is possible that the disease process burned itself out, it seems unlikely in a patient who had been refractory for years with actively increasing inflammation on serial MRIs immediately prior to the initiation of thalidomide. Also, the patient had complex partial seizures, and seizures are another known association with a typically refractory course [9]. Initiation of more aggressive immunosuppressive agents than steroids was attempted for 6 months with imuran, two courses of intravenous cyclophosphamide, and for 7 months with cyclosporine prior to initiation of thalidomide. Even though these medications can take months to show clinical benefit, it is unlikely that the patient would have eventually responded, since she was still progressing after 6 months of therapy. Nevertheless, it is true that the patient only received two full courses of intravenous cyclophosphamide, and the dose of cyclosporine was lower than target doses for treatment in sarcoidosis. At a total dose of 50 mg per day for the cyclosporine (25 mg po BID), this is lower than the initial dose range of up to 4–6 mg/kg/day divided up BID quoted in the literature for use in neurosarcoidosis [4]. So, even though it seems likely that the patient had a true failure of conventional immunosuppressive treatment, due to the above extenuating circumstances, we are only be able to firmly conclude that the patient had neurosarcoidosis refractory to high dose corticosteroids.

Nevertheless, thalidomide yielded a dramatic response in the current patient. We recommend further clinical evaluation of this medication for treatment of patients with neurosarcoidosis that are refractory to corticosteroids and other conventional immunosuppressive therapy.

## Conclusion

Our case report presents a difficult, refractory case of neurosarcoidosis that demonstrates an altered prognosis based on the addition of thalidomide.

## Abbreviations

OD – right eye, OS – left eye, MRI – Magnetic Resonance Imaging, CT – Cat Scan, MHC-II – Major Histocompatibility-II, IFN-γ – interferon-gamma, TNF-α – Tumor necrosis factor-alpha, MS – Multiple Sclerosis, ACE – angiotensin converting enzyme, QD – daily, QOD – every other day, BID – twice daily, TH-1 – T helper-1, STEPS (System for Thalidomide Education and Prescribing Safety)

## Competing interests

The author(s) declare that they have no competing interests.

## Authors' contributions

JCH reviewed the patient's records, the pertinent literature, and drafted the manuscript. HN provided the case report, reviewed the literature, and edited/finalized the manuscript. SK reviewed and finalized the manuscript.

## Consent

Written informed patient consent was obtained for publication of the report and any accompanying images.
